# Weakly-supervised deep learning for ultrasound diagnosis of breast cancer

**DOI:** 10.1038/s41598-021-03806-7

**Published:** 2021-12-21

**Authors:** Jaeil Kim, Hye Jung Kim, Chanho Kim, Jin Hwa Lee, Keum Won Kim, Young Mi Park, Hye Won Kim, So Yeon Ki, You Me Kim, Won Hwa Kim

**Affiliations:** 1 School of Computer Science and Engineering, Kyungpook National University, Daegu, Republic of Korea; 2 Department of Radiology, School of Medicine, Kyungpook National University, Kyungpook National University Chilgok Hospital, Daegu, Republic of Korea; 3 Department of Radiology, Dong-A University College of Medicine, Busan, Republic of Korea; 4 Departments of Radiology, School of Medicine, Konyang University, Konyang Univeristy Hospital, Daejeon, Republic of Korea; 5 Department of Radiology, School of Medicine, Inje University, Busan Paik Hospital, Busan, Republic of Korea; 6 Department of Radiology, Wonkwang University Hospital, Wonkwang University School of Medicine, Iksan, Republic of Korea; 7 Department of Radiology, School of Medicine, Chonnam National University, Chonnam National University Hwasun Hospital, Hwasun, Republic of Korea; 8 Department of Radiology, School of Medicine, Dankook University, Dankook University Hospital, Cheonan, Republic of Korea

**Keywords:** Ultrasonography, Breast cancer, Cancer, Health care, Oncology

## Abstract

Conventional deep learning (DL) algorithm requires full supervision of annotating the region of interest (ROI) that is laborious and often biased. We aimed to develop a weakly-supervised DL algorithm that diagnosis breast cancer at ultrasound without image annotation. Weakly-supervised DL algorithms were implemented with three networks (VGG16, ResNet34, and GoogLeNet) and trained using 1000 unannotated US images (500 benign and 500 malignant masses). Two sets of 200 images (100 benign and 100 malignant masses) were used for internal and external validation sets. For comparison with fully-supervised algorithms, ROI annotation was performed manually and automatically. Diagnostic performances were calculated as the area under the receiver operating characteristic curve (AUC). Using the class activation map, we determined how accurately the weakly-supervised DL algorithms localized the breast masses. For internal validation sets, the weakly-supervised DL algorithms achieved excellent diagnostic performances, with AUC values of 0.92–0.96, which were not statistically different (all *P*s > 0.05) from those of fully-supervised DL algorithms with either manual or automated ROI annotation (AUC, 0.92–0.96). For external validation sets, the weakly-supervised DL algorithms achieved AUC values of 0.86–0.90, which were not statistically different (*P*s > 0.05) or higher (*P* = 0.04, VGG16 with automated ROI annotation) from those of fully-supervised DL algorithms (AUC, 0.84–0.92). In internal and external validation sets, weakly-supervised algorithms could localize 100% of malignant masses, except for ResNet34 (98%). The weakly-supervised DL algorithms developed in the present study were feasible for US diagnosis of breast cancer with well-performing localization and differential diagnosis.

## Introduction

Ultrasound (US) is the mainstay of differential diagnosis between benign and malignant breast masses and has traditionally been used in diagnostic settings with renewed interest of its use in screening settings^1,2^. Despite such wide applicability, breast US has intrinsic limitations, including interobserver variability in diagnostic performance that is often worse among non-experts^3^. This interobserver variability contributes to a high rate of false-positives, causing unnecessary biopsies and surgeries. With expectations to overcome these limitations, there has been a growing interest in the application of deep learning (DL) technology for breast US diagnosis^4–6^. Conventional approaches using DL algorithms have involved full supervision that require image annotation processes usually performed by drawing the region of interest (ROI) of the lesion by humans. Even with automated ROI segmentation methods, verification of ROI by humans is still needed. As DL is a data-driven technology, time- and labor-intensive image annotation process may hinder the development of well-performing models due to the need of massive training data. Moreover, manual annotation can be biased as this task necessarily involves subjective pre-judgment of the lesion.

DL with weak supervision (weakly-supervised DL) is a form of DL where unannotated images with only image-level labels (i.e., malignant and benign) are used in training for differential diagnosis and localization^7–9^. Weakly-supervised DL has advantages over fully-supervised DL approaches in the development of DL algorithm and its clinical application. For developing DL-based algorithm, a method without image annotation can compile large-scale image sets in a time- and labor-saving manner. For clinical application, weakly-supervised DL algorithms allow us to use the entire image as input to the trained model, leading to an improvement in workflow efficiency over fully-supervised algorithms as the additional task of marking lesions can be avoided. Despite these benefits of weakly-supervised DL algorithms, only a few studies have demonstrated their feasibility in radiology. Weakly-supervised DL algorithm was evaluated in magnetic resonance imaging (MRI) or chest x-ray images and demonstrated good diagnostic performances in the classification of breast lesions and thoracic disease^10,11^. However, weakly-supervised DL algorithm has not been well studied in breast US images.

The main hypothesis of this work is that weakly-supervised DL algorithms for US images are feasible for diagnosing breast masses and comparable to conventional fully-supervised DL algorithms. The purpose of this study was to develop a weakly-supervised DL algorithm that detects breast masses in US images and make a differential diagnosis between benignity and malignancy synchronously.

## Material and methods

Institutional review board (IRB) of Kyungpook National University Chilgok Hospital approved this retrospective study and all methods were carried out in accordance with relevant guidelines and regulations. The requirement of informed consent was waived under the IRB of Kyungpook National University Chilgok Hospital.

### Datasets

**Figure 1:**
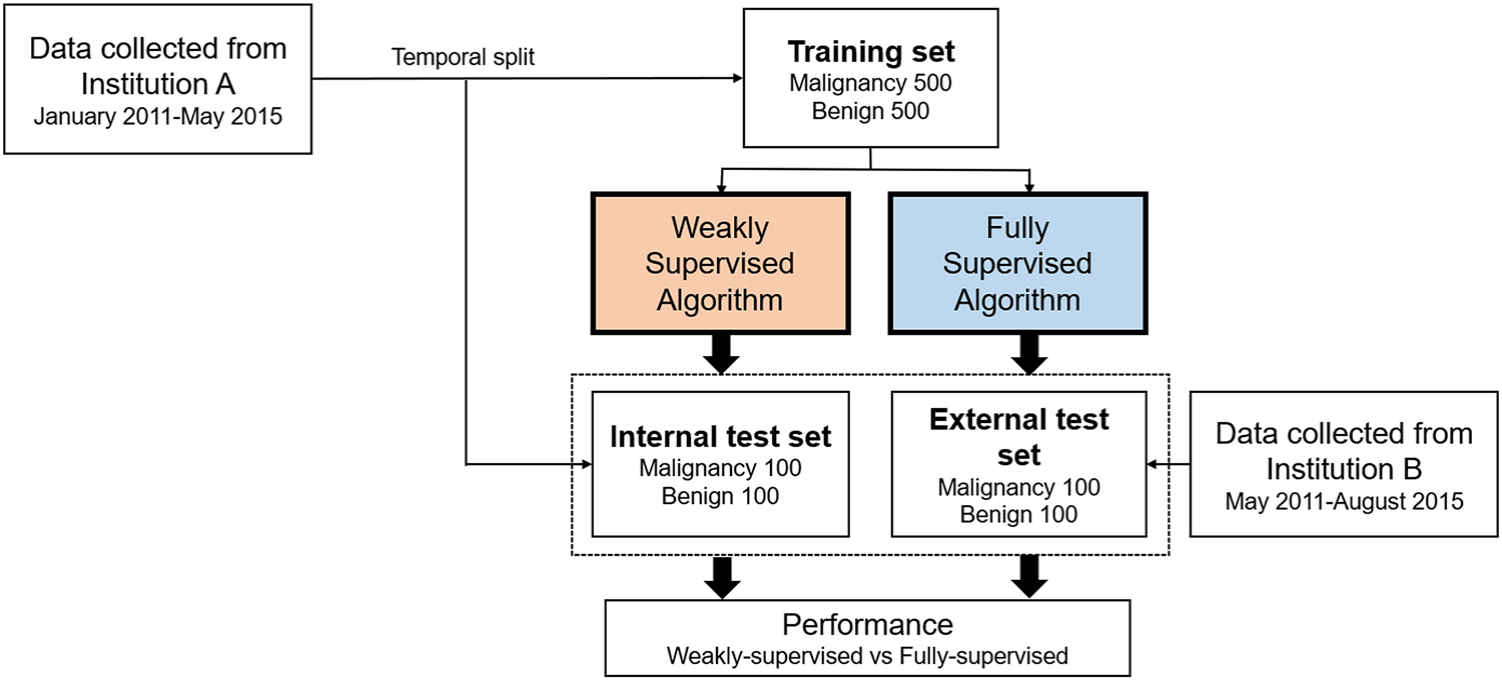
Overview of the data acquisition.

We retrospectively collected 1400 US images for breast masses of 971 patients from two institutions (institution A: A University A′ Hospital; institution B: B University Hospital; Fig. 1) for training and validation sets. Although multiple masses per patient were allowed, the most representative image per mass (usually showing the largest slice of a mass) was used. Among the 1400 images, 700 were images with cancers confirmed by biopsy or surgery, and 700 were images with benign masses that were confirmed by biopsy (n = 163) or at least 2 years of follow-up imaging (n = 537). The training set contained 500 benign and 500 malignant masses obtained from institution A (data collection period: January 2011–May 2015). The validation sets were divided into internal and external validation sets, each with 200 images of 100 benign and 100 malignant masses. Images for internal validation were temporally split from institution A (data collection period: September 2013–July 2014) and were not used for algorithm training. Images for external validation were consecutively obtained from institution B (data collection period: May 2011–August 2015). All breast US images were extracted from picture archiving and communication systems and were stored in JPEG format. For the training and internal validation sets obtained from institution A, only one US equipment manufactured by Philips was used to generate images, while multiple US machines manufactured by Philips, GE, and Siemens were used for the external validation set (obtained from institution B).

### Image annotation and preprocessing

**Figure 2:**
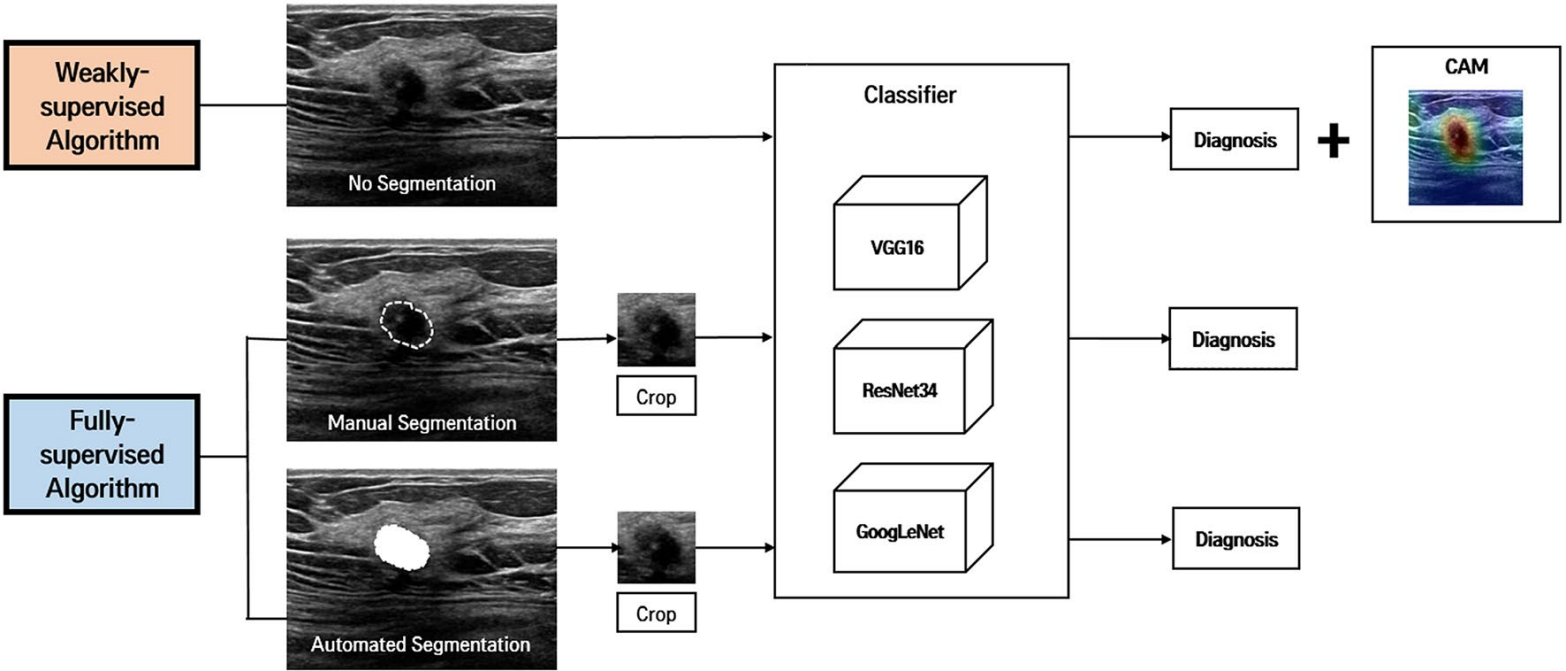
Overview of weakly-supervised and fully-supervised deep learning (DL) algorithms for breast mass classification and localization. The weakly-supervised DL algorithm does not require image annotation of region of interest (ROI) of the lesion, whereas the fully-supervised DL algorithm requires tumor segmentation (manual or automated) and cropping for ROI before being put in the classifiers. For the weakly-supervised DL algorithm, a class activation map (CAM) is generated to visualize the region detected by this algorithm using a global average pooling layer (GAP) that is added to the final convolutional layer.

Images were anonymized by minimal trimming of the edge of images to eliminate body mark and text annotation. For the weakly-supervised DL algorithms, further data curation was not performed to test the feasibility of the proposed system without ROI annotation (Fig. 2). The anonymized images were resized to 224 × 224 pixels for the weakly-supervised DL. Then, their pixel values were normalized to the range of [0, 1] by dividing them the maximum intensity value. For comparison with fully-supervised DL algorithms, ROI annotation was performed using two methods: manual drawing and automated DL-based segmentation. For manual drawing, a radiologist (W.H.K.; with 11 years of experience in breast US) marked ROIs and made binary masks for each mass using an in-house drawing tool. For the automated DL-based segmentation, we employed the deep segmentation network U-Net, which has been developed to segment medical images^12^. After the ROI annotation, we extracted a square image with a fixed margin of 30 pixels that enclosed the corresponding mass, resized the image to 224 × 224 pixels, and the pixel intensity normalization using the maximum value was applied.

### Deep classification models

For deep classifiers, we employed three representative convolutional neural networks (CNN) that have achieved the state-of-the-art performance in various computer vision tasks: VGG16, ResNet34, and GoogLeNet^13–15^. Details for each VGG16, ResNet34, and GoogLeNet were given in our Supplemental Digital Content.

To test the performance of the discriminative localization by the weakly-supervised DL algorithms, we extended the classification models using a global average pooling layer (GAP) that is added to the final convolutional layer of each model^10,16^. The GAP averages each feature map ($f_{k}$) of the last convolution layer into feature scores ($F_{k}$) as follows.

$F_{k} = \sum\nolimits_{i, j} {f_{k} \left( {i, j} \right)}$ where $i$ and $j$ are the spatial indices of $f_{k}$. The number of the feature maps is same as that of classes ($N_{c}$). Then, the models perform linear classification using a fully-connected layer followed by a softmax function. The fully-connected layer with learnable weights $\left( {{\text{W}} = \left\{ {w_{k,c} } \right\}} \right)$ calculates class scores ($S_{c}$) for each class as follows.

$$ S_{c} = \mathop \sum \limits_{k} w_{k,c} F_{k} $$

The class score is given to the softmax function to yield the predicted probabilities of all classes. The predicted probability ($p_{c}$) of each class and probability of malignancy (POM) was calculated as follows:

$$ p_{c} = \frac{{{\text{exp}}\left( {S_{c} } \right)}}{{\mathop \sum \nolimits_{k = 1}^{{N_{c} }} {\text{exp}}\left( {S_{k} } \right)}} $$

$$ POM = \left\{ {\begin{array}{*{20}l} {P_{c} , } \hfill & {if\, c\, = \,malignancy} \hfill \\ {1 - P_{c} ,} \hfill & { otherwise} \hfill \\ \end{array} } \right. $$

The neural networks were trained using the L2 regularization, a batch size of 64, and the Adam optimizer with learning rate ($\eta = 0.001$), $\beta = 0.9$, and $\beta_{2} = 0.999$. The learnable weights of the neural networks are initialized the He initialization. For the hyper-parameter selection, we performed grid search using a tuning set (100 for benign and 100 for malignancy), randomly selected from the training set. After the hyper-parameter selection, we trained all networks using the whole training set. The model training and all experiments were conducted on a workstation with NVidia TITAN XP (12 GB Memory), Intel i9 CPU, and 48 GB main memory. The neural networks and algorithms were implemented using Python 3.6.9 and PyTorch 1.1.0.

### Discriminative localization

The class activation maps ($M_{c}$) of each class can be acquired by merging the feature maps using the weights that are learned in the estimation of the class scores.

$$ M_{c} = \mathop \sum \limits_{k} w_{k,c} f_{k} $$

The relative intensity of $M_{c}$ is scaled using a min–max normalization for inter-subject comparison and visualization. The scaled class activation maps ($M_{c}^{^{\prime}}$) are acquired as follows.

$$ M_{c}^{^{\prime}} = \frac{{M_{c} - {\text{min}}\left( {M_{c} } \right)}}{{\max \left( {M_{c} } \right) - {\text{min}}\left( {M_{c} } \right)}} $$

Focused regions in the tumor characterization are determined by the binarization of the scaled maps with a threshold ($M_{c}^{^{\prime}} \ge 0.3$). The threshold was chosen empirically considering the overlap of the binarized maps with the manual ROI annotation in the training set.

### Performance metrics and statistical analysis

For differential diagnosis, we used area under the receiver operating characteristics curve (AUC) as the primary metric for comparing the algorithm performance, and the DeLong test of significance for comparing the AUC of two correlated receiver operating characteristics curves (ROCs). The exact McNemar test was used to test the differences in sensitivity and specificity. Discriminative localization is regarded as correct when the segmented area overlaps with the manually annotated area. Fisher’s-exact test was used to compare rates of correct and incorrect localization between benign and malignant masses with the weakly-supervised DL algorithm. For the automated ROI segmentation performance, the dice similarity coefficient (DSC) was calculated to assess spatial agreement between the automatically segmented ROI ($M_{s}$) and the manual ROI annotation ($M_{g}$):

$$ {\text{DSC}} = \frac{{2\left| {M_{s} \cap M_{g} } \right|}}{{\left| {M_{s} } \right| + \left| {M_{g} } \right|}}, $$

where $\left| {M_{s} } \right|$ and $\left| {M_{g} } \right|$ are the number of pixels of the automated and manual ROIs, respectively. $M_{s} \cap M_{g}$ represents common regions to both of ROIs. All statistical analyses were performed using the MedCalc statistical software, version 17.1 (Mariakerke, Belgium). Two-tailed *P* values of < 0.05 were considered statistically significant.

## Results

### Baseline characteristics of data sets

Baseline characteristics of the training set and internal/external validation sets are described in Table 1. The mean ages of patients in training, internal validation, and external validation sets were 49 years (range: 19–83 years), 50 years (range: 22–79 years), and 50 years (range: 16–80 years), respectively. The mean sizes of the lesions measured via US were 15 mm (range: 1–69 mm), 14 mm (range: 5–60 mm), and 15 mm (range: 6–47 mm), respectively.

**Table 1:** Baseline characteristics of datasets.

	Training	Internal validation	External validation
Patients	818	167	125
Images	1000	200	200
Patients with malignant masses	500	100	88
Images with malignant masses	500	100	100
Patients with benign masses	346	68	39
Images with benign masses	500	100	100
Age groups*			
< 30 years	16 (2%)	3 (2%)	12 (6%)
30–50 years	431 (53%)	93 (56%)	91 (46%)
≥ 50 years	371 (45%)	71 (43%)	97 (49%)
Tissue composition**			
Homogeneous, fat	20 (2%)	5 (3%)	14 (11%)
Homogeneous, fibroglandular	956 (96%)	191 (96%)	69 (55%)
Heterogeneous	24 (2%)	4 (2%)	42 (34%)
Lesion size**			
< 2 cm	754 (75%)	149 (75%)	163 (82%)
2–5 cm	239 (24%)	50 (25%)	37 (19%)
≥ 5 cm	7 (1%)	1 (1%)	0
US machine**			
Phillips	1000 (100%)	200 (100%)	16 (8%)
GE	0	0	169 (85%)
Siemens	0	0	15 (8%)

*Patient-level, **image-level.

### Performance metrics of differential diagnosis

For internal validation test set, the weakly-supervised DL models achieved high performances in the differential diagnosis between benign and malignant breast masses, with AUC values of 0.96, 0.92, and 0.94in VGG16, ResNet34, and GoogLeNet models, respectively (Table 2). The AUCs of fully-supervised DL models with manual annotation were 0.96, 0.94, and 0.96 in VGG16, ResNet34, and GoogLeNet models, respectively. The AUCs of fully-supervised DL models with automated annotation were 0.96, 0.92, and 0.95, respectively. The DSC of the automated ROI annotation for internal validation test set was 0.89 (standard deviation, 0.12). The AUCs of weakly-supervised DL models were not different from those of fully-supervised DL models with either manual or automated ROI annotation (all *P*s > 0.05). Sensitivities of weakly-supervised DL models were 87% (87/100), 82% (82/100), and 87% (87/100) in VGG16, ResNet34, and GoogLeNet models, respectively, and specificities were 91% (91/100), 91% (91/100), and 94% (94/100), respectively. The sensitivities and specificities were not different between weakly-supervised and fully-supervised DL models (all *P*s > 0.05).

**Table 2:** Diagnostic performance metrics of weakly-supervised and fully-supervised deep learning algorithms in internal validation set.

	Weakly-supervised	Fully-supervised		*P* values	
		Manual	Automated	Weakly-supervised vs. manual	Weakly-supervised vs. automated
**AUC**					
VGG16	0.96 (0.92, 0.98)	0.96 (0.93, 0.98)	0.96 (0.92, 0.98)	0.72	0.96
ResNet34	0.92 (0.88, 0.96)	0.94 (0.89, 0.97)	0.92 (0.87, 0.95)	0.57	0.76
GoogLeNet	0.94 (0.90, 0.97)	0.96 (0.92. 0.98)	0.95 (0.91, 0.98)	0.30	0.65
**Sensitivity**					
VGG16	87%	85%	87%	0.77	1.00
ResNet34	82%	89%	79%	0.12	0.63
GoogLeNet	87%	87%	87%	1.00	1.00
**Specificity**					
VGG16	91%	91%	94%	1.00	0.45
ResNet34	91%	90%	92%	1.00	1.00
GoogLeNet	94%	92%	92%	0.69	0.69

95% confidence intervals in parenthesis.

**Figure 3:**
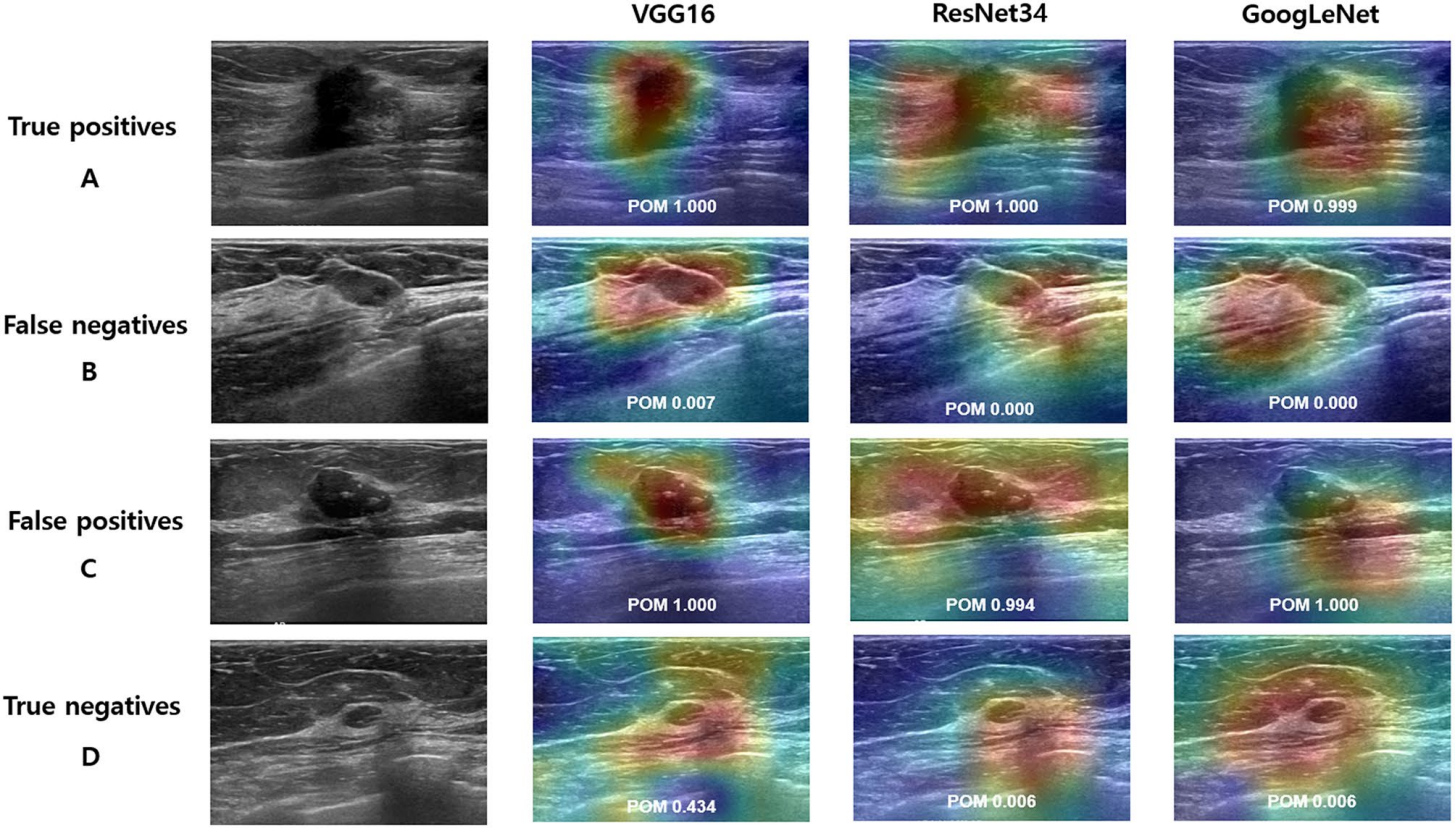
Classification results with class activation map (CAM) using the weakly-supervised deep learning (DL) algorithm on external validation set. Examples of true-positive (**A**), false-negative (**B**), false-positive (**C**), and true-negative (**D**) are shown for each network (VGG16, ResNet34, and GoogLeNet). (**A**) Ultrasound images show a 17-mm irregular, spiculated invasive ductal carcinoma, which was predicted as malignancy with probability of malignancy (POM) of 1.00, 1.00, and 0.999 in VGG16, ResNet34, and GoogLeNet, respectively. (**B**) Ultrasound images show an 11-mm oval, circumscribed, isoechoic mucinous carcinoma, which was predicted as benign with POM of 0.007, 0.000, and 0.000, respectively. (**C**) Ultrasound images show a 29-mm oval, hypoechoic mass with macrocalcifications considered as benign (unchanged during the 46-month follow-up period), which was predicted as malignancy with POM of 1.000, 0.994, and 1.000, respectively. (**D**) Ultrasound images show a 6-mm oval, circumscribed mass considered as benign (unchanged during the 55-month follow-up period), which was predicted as benign with POM of 0.434, 0.006, and 0.006, respectively.

For external validation test set, the weakly-supervised DL model achieved high diagnostic performance but was slightly lower than those in internal validation sets, with AUC values of 0.89, 0.86, and 0.90 in VGG16, ResNet34, and GoogLeNet models, respectively (Table 3, Fig. 3). The AUCs of fully-supervised DL models with manual annotation were 0.91, 0.89, and 0.92 in VGG16, ResNet34, and GoogLeNet models, respectively. The AUCs of fully-supervised DL models with automated annotation were 0.85, 0.84, and 0.87, respectively. The DSC of the automated ROI annotation for external validation test set was 0.85 (standard deviation, 0.17). The AUCs of weakly-supervised DL models were not statistically different from those of fully-supervised DL models with manual ROI annotation (all *P*s > 0.05). For the VGG16 network, the AUC was significantly higher in the weakly-supervised DL model than the fully-supervised DL model with automated ROI annotation (*P* = 0.04). ResNet34 and GoogLeNet networks showed no significant differences between weakly-supervised DL model and fully-supervised DL model with automated ROI annotation (all *P*s > 0.05). Sensitivities of weakly-supervised DL models were 91% (91/100), 78% (78/100), and 88% (88/100) in VGG16, ResNet34, and GoogLeNet models, respectively, and the specificities were 72% (72/100), 80% (80/100), and 76% (76/100), respectively. The sensitivities did not significantly differ between weakly-supervised and fully-supervised DL models in VGG16 and GoogLeNet (all *P*s > 0.05). For the ResNet34 model, the sensitivity was lower in the weakly-supervised DL model than the fully-supervised model with manual annotation (*P* < 0.001) but not significantly different from the fully-supervised DL model with automated ROI annotation (*P* = 0.66). The specificity of the weakly-supervised DL model was not significantly different from that of fully-supervised DL models with manual ROI annotation in VGG16 and GoogLeNet models (all *P*s > 0.05) and lower than that in the ResNet34 model (*P* < 0.001). The specificity was higher in the weakly-supervised DL model than the fully-supervised DL model with automated ROI annotation with statistical significance or borderline significance (*P* < 0.001, *P* = 0.07, and *P* = 0.04 in VGG16, ResNet34, and GoogLeNet models, respectively).

**Table 3:** Diagnostic performance metrics of weakly-supervised and fully-supervised deep learning algorithms in external validation set.

	Weakly-supervised	Fully-supervised		*P* values	
		Manual	Automated	Weakly-supervised vs. manual	Weakly-supervised vs. automated
**AUC**					
VGG16	0.89 (0.84, 0.93)	0.91 (0.86, 0.95)	0.85 (0.79, 0.89)	0.28	0.04
ResNet34	0.86 (0.81, 0.91)	0.89 (0.84, 0.93)	0.84 (0.78, 0.88)	0.31	0.32
GoogLeNet	0.90 (0.85, 0.94)	0.92 (0.87, 0.95)	0.87 (0.82, 0.92)	0.32	0.19
**Sensitivity**					
VGG16	91%	85%	89%	0.45	0.73
ResNet34	78%	89%	81%	< 0.001	0.66
GoogLeNet	88%	87%	87%	0.51	1.00
**Specificity**					
VGG16	72%	91%	52%	0.85	< 0.001
ResNet34	80%	90%	69%	< 0.05	0.07
GoogLeNet	76%	92%	63%	0.85	0.04

95% confidence intervals in parenthesis.

### Performance metrics of discriminative localization

In internal validation sets, the weakly-supervised DL model using VGG16 and ResNet34 network could localize 99% (99/100) of the benign and 100% (100/100) of malignant masses (Table 4). The weakly-supervised DL model using the GoogLeNet network could localize 100% (100/100) of both benign and malignant masses. In external validation sets, the weakly-supervised DL model using VGG16, ResNet34, and GoogLeNet networks could localize 99% (99/100), 96% (96/100), 97% (97/100) of the benign, and 100% (100/100), 98% (98/100), 100% (100/100) of malignant masses, respectively.

**Table 4:** Metrics for discriminative localization of benign and malignant breast masses in the weakly-supervised deep learning algorithm.

	Internal validation			External validation		
	Benign	Malignant	*P* value	Benign	Malignant	*P* value
VGG16			1.00			1.00
Correct	99	100		99	100	
Incorrect	1	0		1	0	
ResNet34			1.00			0.68
Correct	99	100		96	98	
Incorrect	1	0		4	2	
GoogLeNet			N/A			0.25
Correct	100	100		97	100	
Incorrect	0	0		3	0	

N/A = not applicable. For discriminative localization, we created binary images by applying a threshold of 0.3 to a class activation map (CAM) and compared them with manual annotation. Discriminative localization is regarded as correct when the segmented area overlaps with the manually annotated area.

## Discussion

In this study, we found that weakly-supervised DL algorithm provided excellent diagnostic performance (AUC: 0.86–0.96) that were not inferior to the fully-supervised DL algorithm with manual (AUC: 0.89–0.96) and automated annotation (AUC: 0.84–0.96). Furthermore, the weakly-supervised DL algorithm could correctly localize the benign and malignant masses with nearly perfect rates (96%–100%). This excellent classification and localization performance was achieved even in our relatively small-sized dataset and in the external validation set with different breast imagers and US equipment. Taken together, our results suggests that weakly-supervised DL algorithm is feasible to detect and diagnose breast cancer in US images through a highly efficient data-curation process in which image-based classification can be made without manual or automated annotation.

Classification methods using the DL algorithm can be categorized into region- and image-based classification. Most of previous studies have used region-based classification. Regions (usually for lesions) are necessarily determined prior to classification task either manually (including semi-automatically) or automatically. Studies using manually determined regions showed high diagnostic performances with AUCs 0.84–0.94 depending on cases and strategies for learning^17–19^. A commercial computer-aided diagnosis (CAD) system (S-detect) that is installed in certain US equipment (RS80A, Samsung Medison Co. Ltd., Seoul, South Korea) enables semi-automated region annotation and predicts the final assessment in a dichotomized form (possibly benign or possibly malignant). With this system, Park et al. found that AUC was improved with CAD (0.823–0.839 vs. 0.623–0.759), especially for less-experienced radiologists^20^. In other studies, regions were determined more inclusively by cropping the image by human with excellent diagnostic performances (AUC, 0.913 and 0.951)^21,22^. Automated determination of regions has been proposed using various methods of region proposals. Diagnostic performances using the automatically determined regions were suboptimal or metrics were not-well demonstrated with the highest accuracy of 87.5% in a study using DenseNet^13^, 60.6% of sensitivity for malignant lesions in a study using fully convolutional networks^23^, no overall diagnostic metrics in a study using faster R-CNN^24^. Image-based classification with weakly-supervised DL algorithms has been proposed in the present study and our previous work^25^. In our previous work, we proposed a box convolution network with VGG-16 which learns kernel sizes and offsets of convolution filters from given datasets. We found that our proposed model had higher performances in diagnostic accuracy and localization than VGG-16 or dilated VGG-16. While our previous work was focused on box convolution network, we did not compare our model with fully-supervised DL algorithms and external validation or generalization to other networks were not evaluated. In the present study, using three representative networks and external validation test sets, the feasibility of weakly-supervised DL algorithm was demonstrated in comparison with a fully-supervised DL algorithm.

Weakly-supervised DL algorithms serve more closely as a human-mimicking algorithm than fully-supervised DL algorithm in differentiating malignant masses from benign breast masses in US images. Human-established algorithms employed in breast imaging reporting and data system (BI-RADS) take into account comprehensive sonographic features of both the mass and the surrounding breast tissue. Hence, weakly-supervised DL, using the information of the images in their entirety (not confined to the mass or its vicinity), may have advantages over fully-supervised DL; the proposed algorithm can learn a significant portion of BI-RADS lexicon describing information outside the mass (e.g., posterior features, architectural distortion) that is known to be helpful for differential diagnosis^26,27^.

For the fully-supervised DL as a comparative method, we implemented a U-Net based segmentation model as a baseline method of the automated segmentation of breast US lesions. Automated segmentation has been suggested in various techniques including graph-based approaches, deformable models, intensity-based thresholding, region growing, and watershed algorithm^28^. Recently, DL techniques including U-Net architecture have shown to achieve good performances on various biomedical segmentation tasks^29^. In breast US, several variants of the U-Net for mass segmentation have been proposed. Yap, et al. demonstrated that their performing model based on fully convolutional networks obtained 0.76 and 0.55 of DSCs for benign and malignant breast masses, respectively^30^. Byra, et al. proposed selective kernel U-Net model and between performances were achieved (0.80 and 0.74 for benign and malignant breast masses, respectively) using the sample datasets^31^. Hu, et al. developed a DL method using dilated fully CNN combined with an active contour model and their best performance was 0.89 of DSC based on 570 ultrasound images^32^. Although it is difficult to compare our results with those reported due to different methods and evaluation schemes, our U-Net performances based on 1000 US images were comparable with the previous reports (0.89 and 0.85 for internal and external validation sets). However, we did not aim to propose the state-of-the-art segmentation method and U-Net was used as a basic example of the automated segmentation for fully-supervised method in comparison with weakly-supervised method and fully-supervised method with manual annotation as a lesion ground-truth was also used in our study.

GAP used in our study can enforce the feature maps to preserve spatial information relevant to the classes, so that they can be used to interpret the decision of the CNN models^8,33^. This method for identifying areas that are attributed to differential diagnosis using GAP with CAM leads toward the concept of eXplainable AI (XAI)^34,35^. XAI or responsible AI is an emerging paradigm to overcome the inherent “black box problem” brought by deep frameworks, wherein it is impossible for us to understand how decisions are furnished. CAM as one of the feature attribution maps give us an insight to interpret the decision-making process implemented by AI. In addition, we believe that the weakly-supervised DL with CAM may facilitate the development of DL-aided detection frameworks for clinically significant regions for healthcare providers^36,37^. For the interpretability of the DL models, various feature attribution methods have been proposed in three categories^38^: gradient-based approaches (e.g., Grad-CAM and Layer-wise relevance propagation), perturbation-based approaches, and surrogate models (e.g. LIME and SHAP). For example, as proposed in previous study, approximation a surrogate model between input image features and the model’s classification output for computation Shapely values, which represent the contribution of each feature to the output, may enable us to recognize image regions relevant to the decision-making process of the classification models^39^.

An important caveat in the present study was that our proposed weakly-supervised DL algorithm was not trained with a large-scale dataset due to our feasibility objectives. Further studies are needed using dataset with various institutions, imagers, and US equipment. Another limitation is that a time- and labor-efficiency was not directly quantified because of the complexity of data curation process.

In summary, we have developed a weakly-supervised DL algorithm without image ROI annotation to detect and diagnose breast masses, suggesting a highly efficient algorithm in data curation. The proposed algorithm showed comparable performance to fully-supervised DL algorithms for differential diagnosis and localization.

## Supplementary Information

Supplementary Information.

## Abbreviations

DL: Deep learning
ROI: Region of interest
CAM: Class activation map
CNN: Convolutional neural networks
GAP: Global average pooling
POM: Probability of malignancy
AUC: Area under the receiver operating characteristics curve
ROC: Receiver operating characteristics curve
CI: Confidence interval
CAD: Computer-aided diagnosis
