# Supplementary material for: Weakly-supervised deep learning for ultrasound diagnosis of breast cancer
Source: Sci Rep. 2021 Dec 21;11:24382. doi: 10.1038/s41598-021-03806-7 (PMC8692405; doi:10.1038/s41598-021-03806-7)

**Supplemental Digital Content**

***Deep Classification Models***

The VGG16 is a feedforward neural network that stacks five convolutional blocks, where each block consists of two 3$\times$3 convolutional layers followed by a max-pooling layer, to learn relevant image features for the classification task. The ResNet34 architecture is a deeper CNN with a residual connection that can solve the vanishing gradient problem as a short cut after weight layers. GoogLeNet is composed of stacks of Inception modules to learn visual patterns for different scales. A single Inception module comprises multiple convolution branches with different filter sizes, ranging from 1$\times$1 to 5$\times$5, to augment receptive fields in a cost-efficient manner.

***VGG16***

VGG16 has a simple structure that consists of convolutional layers using 3x3 kernel, max-pooling layers after some convolutional layers, and fully-connected layers as a classifier. In this paper, we modified VGG16 for the purpose of this research. We replaced the ordinary classifier that uses fully-connected layers into the global average pooling layer that keep spatial information during classification task. As a result, we can compute GAP (global average pooling) that detects the tumor region in US images and keep the classification performance. Also, we removed the last pooling layer to extend the mapping resolution, which improves the localization ability of the network. And, we applied batch normalization to the output of the convolutional layer to help the network converge early and prevent vanishing gradient problem. Table 1 has shown the architecture of VGG16.

***ResNet34***

ResNet34 uses residual learning to stack more hidden layers. Conventional networks aim to get optimal output through learning. But, residual learning aims to learn the difference between the input and the output by connecting a shortcut after the weight layer. It simplifies the forward and backward path by connecting the input and the output through several layers, which makes it easy to optimize even deeper network and improve accuracy because of increased depth. ResNet34 has residual blocks with a shortcut connection after two convolutional layers, as shown in Figure 1. Most of the convolutional layer uses 3x3 kernels. When reducing the size of the feature-map, it uses stride as 2 not pooling (except the first pooling). After this, ResNet34 classifies breast cancer through the global average pooling layer and softmax function. See Table 2 for detailed architecture.

***GoogLeNet***

GoogLeNet uses the inception module shown in Figure 2 to reduce dimension effectively, which is important to make the network deeper and reduce parameters. The inception module uses various size filters in the same layer to extracts more various features in the local receptive field. Also, it uses 1x1 convolution operations so that it can reduce dimension and computation. GoogLeNet uses 9 inception modules. In the inception module, 1x1, 3x3, and 5x5 convolution and 3x3 max-pooling were placed in parallel and 1x1 convolution was used to reduce the number of feature-map. After inception module, the network compressed each feature-map through the global average pooling layer and classifies breast cancer by applying softmax function. The table 3 represents the GoogLeNet architecture.

***Global Average Pooling***

A global average pooling layer averages each feature-map into a single value at the last convolutional layer. Using these values, the network can predict breast cancer. Also, the global average pooling layer takes two advantages. The first is that it can solve the overfitting problem and significantly reduce computation and parameters. Because it can act as a classifier by replacing the fully-connected layer. Another one is that unlike the fully-connected layer, it can keep spatial information, which makes us know how much each feature-map contribute to prediction. Like the weighted sum of average values is the final output of the network, we can obtain GAP that detects the discriminative region by calculating the weighted sum of feature-maps. The highlighted region in the class activation map means the tumor in US images.

***Cross-Entropy Loss***

We trained the classification models (VGG16, GoogLeNet, ResNet34) using cross-entropy loss function (CE) with softmax function. In the classification task, the breast cancer is represented as a one-hot encoded vector in which the true class is set to 1 and 0 for the other classes. Using that vector, CE compares the actual distribution and the predicted distribution. Through optimization, the model gradually outputs predictions similar to the true class to reduce the loss.

***U-Net***

U-Net has been developed to segment more accurately with fewer image data. It consists of a contracting path to shrink the image and an expanding path to grow the image again. The contracting path extracts the context of the image and consists of two convolutional layers using 3x3 kernel and 4x4 convolutional layers with stride as 2 for down-sampling. The expanding path up-samples the feature-map using up-convolution and concatenates this with the context captured in the contracting path for better localization. To do this, U-Net adds skip connections in the encoder-decoder structure. Through the skip connection, more accurate prediction is possible by maintaining detailed pixel information lost during the down-sampling and up-sampling process. In the last layer, a 1x1 convolution converts the feature-maps to a binary mask. To train U-Net, we used mean squared error loss function (MSE) with a sigmoid activation function. MSE minimizes the difference between the ground truth and the output of U-Net. In other words, it makes the U-Net’s output detect the tumor in US images. The U-Net architecture have been detailed in Table 4.

***Fully-supervised Model with Automatic Mass ROIs***

For the automatic ROI annotation, we employed U-Net that has been developed to segment medical images with supervision using manual annotation as labels. It extracts the context of images and reduces the resolution of images using convolutional layers with stride as 2 through the contracting path. And then, it upsamples feature maps via transposed convolution operations to the size of the input. What distinguishes U-Net from the ordinary encoder-decoder structure is that it concatenates the context captured during downsampling with corresponding feature maps at every step in the expanding path. Through the skip connection, more accurate prediction is possible by maintaining detailed pixel information lost during the downsampling and upsampling process. In the last layer, a 1x1 convolution operation converts feature maps into a one-channel image. To train the network, we used mean squared error loss function (MSE) with the sigmoid activation function. MSE minimizes the difference between the output of U-Net and the ground truth. In other words, it makes the U-Net detect the tumor in US images. Since the result from U-Net has continuous values that have passed through the sigmoid function, we set a threshold as 0.6 to turn it into a binary image like manual annotation. Base on this image, we cropped the tumor with a margin of 30 pixels. The rest of the process is the same as that of the manual annotation.

Table S1. Architecture of VGG16.


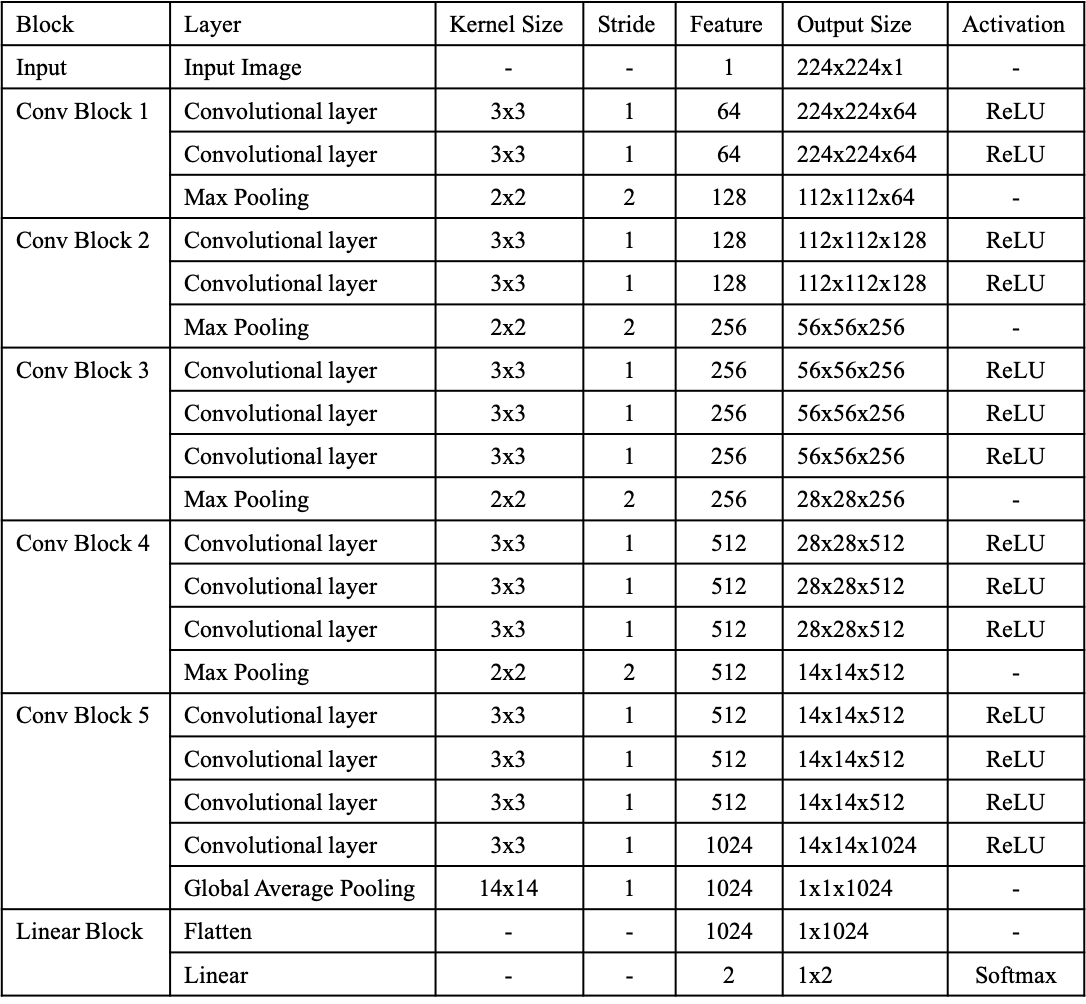


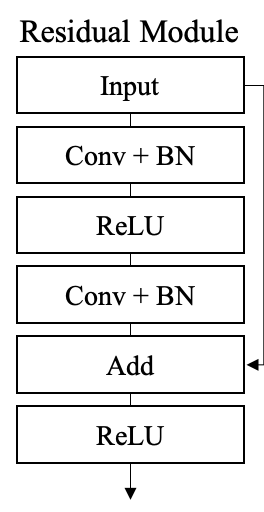


Figure S1. A diagram of a residual module used in ResNet34.

Table S2. Architecture of ResNet34.


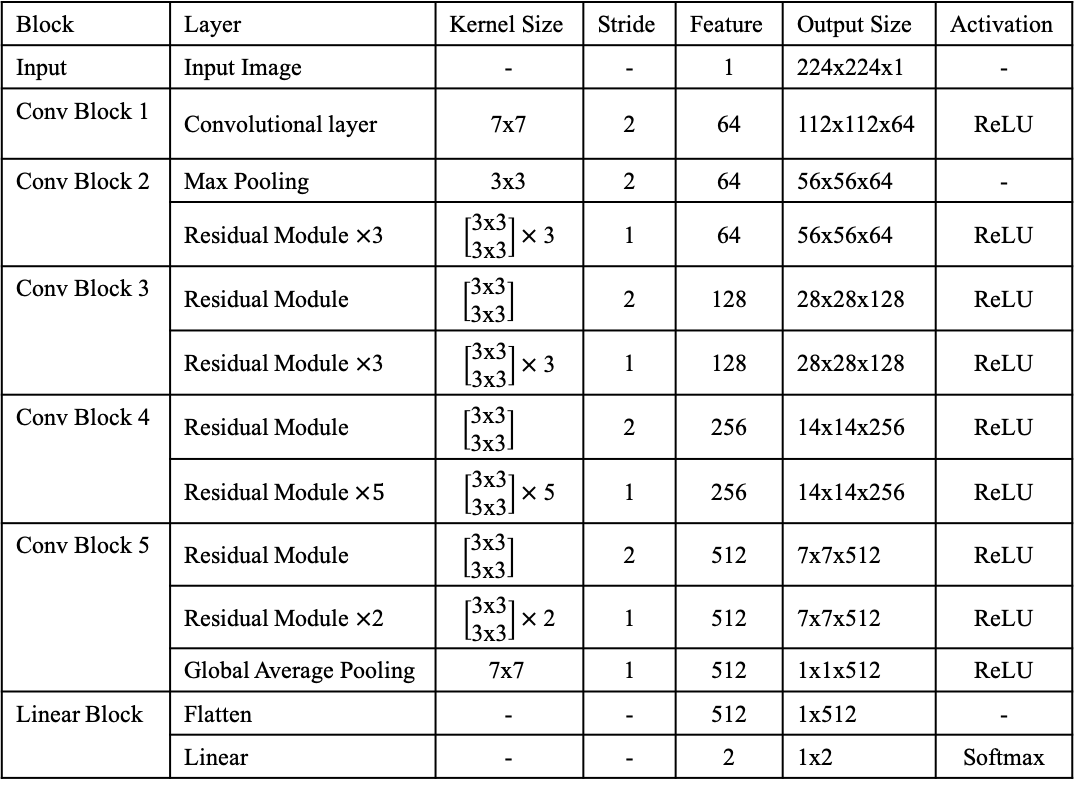


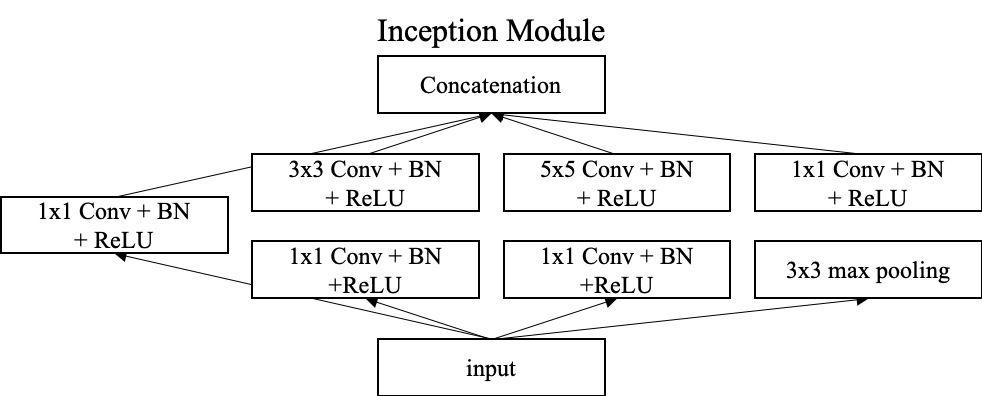


Figure S2. A diagram of Inception module used in GoogLeNet.

Table S3. Architecture of GoogLeNet.


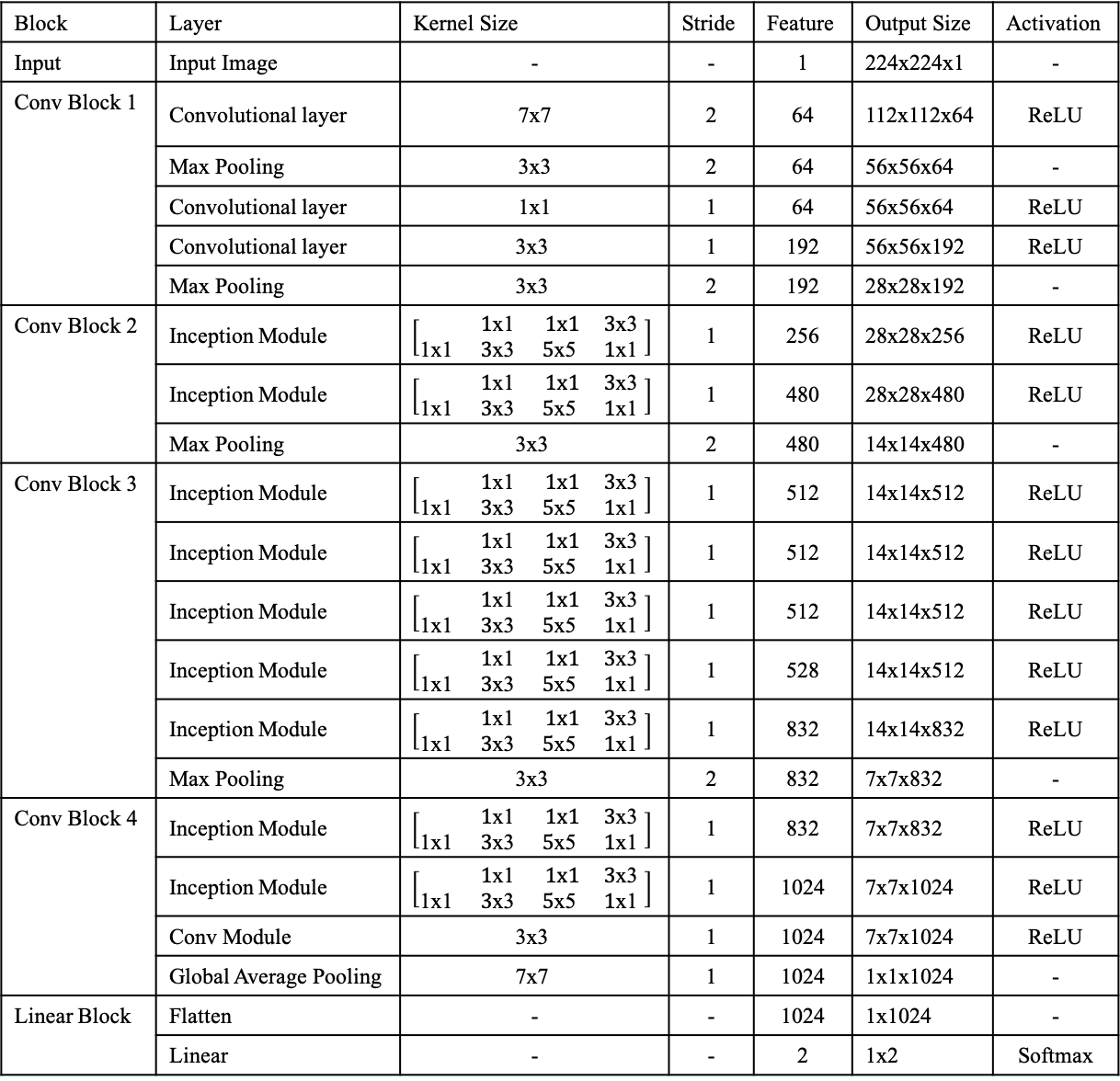


Table S4. Architecture of U-Net.


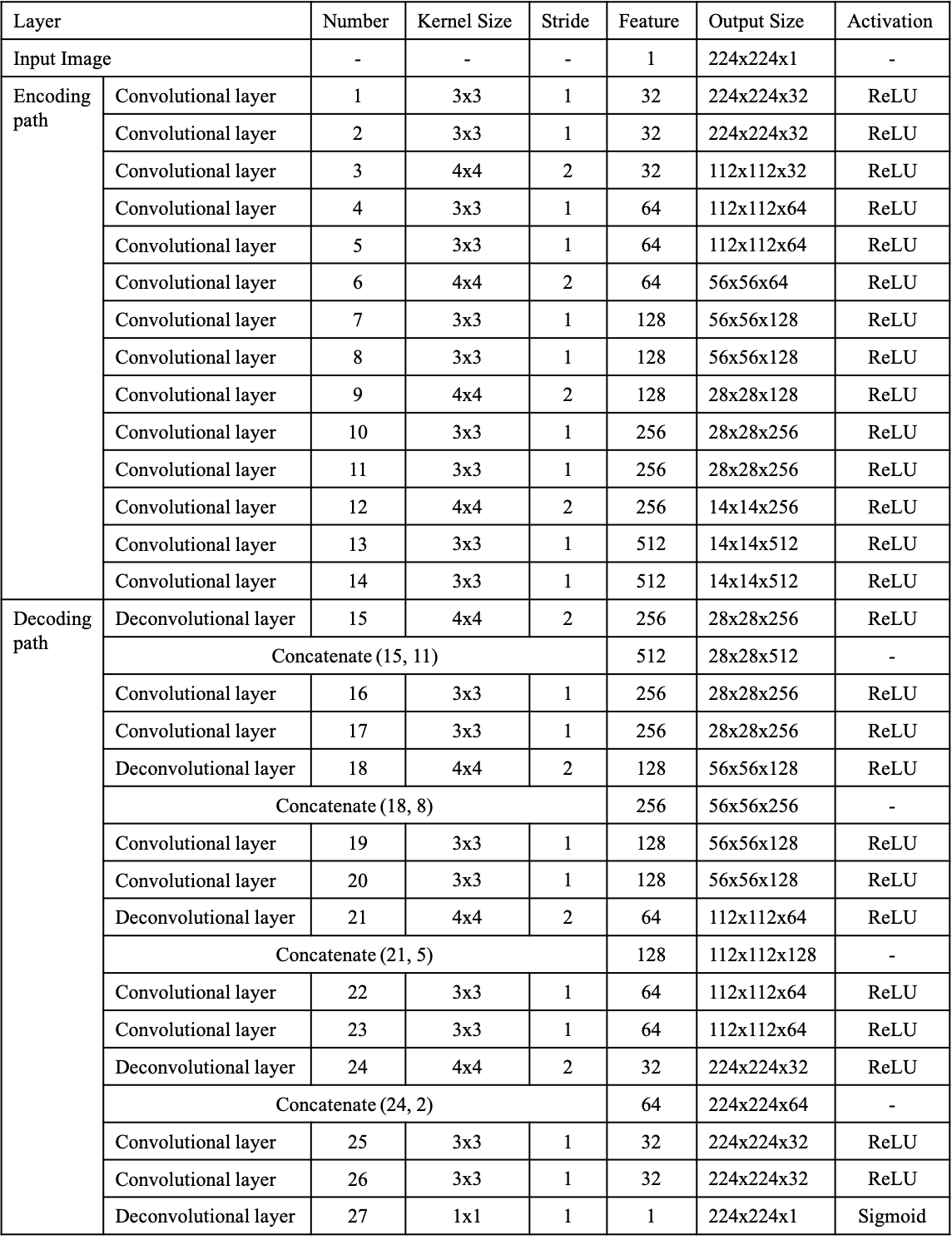

Supplement: Supplementary file 1 — Supplementary Information. [file 41598_2021_3806_MOESM1_ESM.docx]
